# PTR-MS in Italy: A Multipurpose Sensor with Applications in Environmental, Agri-Food and Health Science

**DOI:** 10.3390/s130911923

**Published:** 2013-09-09

**Authors:** Luca Cappellin, Francesco Loreto, Eugenio Aprea, Andrea Romano, José Sánchez del Pulgar, Flavia Gasperi, Franco Biasioli

**Affiliations:** 1 Research and Innovation Centre, Fondazione Edmund Mach (FEM), Via E. Mach 1, San Michele all'Adige 38010, Italy; E-Mails: luca.cappellin@fmach.it (L.C.); eugenio.aprea@fmach.it (E.A.); andrea.romano@fmach.it (A.R.); jose.sanchez@fmach.it (J.S.P.); flavia.gasperi@fmach.it (F.G.); franco.biasioli@fmach.it (F.B.); 2 Institute of Agro-Environmental and Forest Biology, National Research Council, Via Salaria km 29, 300-00015 Monterotondo Scalo, Roma, Italy

**Keywords:** PTR-MS, Proton Transfer Reaction-Mass Spectrometry, PTR-ToF-MS, time of flight, Italy

## Abstract

Proton Transfer Reaction Mass Spectrometry (PTR-MS) has evolved in the last decade as a fast and high sensitivity sensor for the real-time monitoring of volatile compounds. Its applications range from environmental sciences to medical sciences, from food technology to bioprocess monitoring. Italian scientists and institutions participated from the very beginning in fundamental and applied research aiming at exploiting the potentialities of this technique and providing relevant methodological advances and new fundamental indications. In this review we describe this activity on the basis of the available literature. The Italian scientific community has been active mostly in food science and technology, plant physiology and environmental studies and also pioneered the applications of the recently released PTR-ToF-MS (Proton Transfer Reaction-Time of Flight-Mass Spectrometry) in food science and in plant physiology. In the very last years new results related to bioprocess monitoring and health science have been published as well. PTR-MS data analysis, particularly in the case of the ToF based version, and the application of advanced chemometrics and data mining are also aspects characterising the activity of the Italian community.

## 1. Introduction

Being released in many biological and technological processes, and being ubiquitous, volatile organic compounds (VOCs) are important in several fields, including environmental and atmospheric chemistry, plant biology, food science and technology, medical sciences.

An efficient monitoring of VOCs, for such wide ranging applications, needs analytical techniques that are capable of dealing with challenging issues: first, the need of separating and quantifying VOCs in complex gas mixtures; second, the need to detect concentrations that may span a large range, from trace levels to parts per million or more; and third, the need to track concentrations that rapidly change in time. Because of these experimental constraints, the ideal methodology for VOC monitoring should be highly selective, with high sensitivity and dynamic range, and with high time resolution. Fulfilling all these requirements is still a challenge.

The most established technique for identification and quantification of VOCs is certainly gas chromatography–mass spectrometry (GC-MS). GC-MS systems have high precision and, if coupled with suitable pre-treatment and pre-concentration stages, can reach detection limits as low as 0.1 pptv. However, GC-MS suffers from a relatively low time resolution and risk of artefacts.

Overcoming such problems almost unavoidably means employing techniques without chromatographic separation. Different methods have been proposed, such as arrays of solid-state gas sensors [1], spectroscopy [2] and direct injection mass spectrometry (DI-MS). The latter in particular is experiencing an increasing success thanks to its stability and to the fact that the signal being measured (*i.e.*, the mass/charge (*m*/*z*) ratio) is an intrinsic property of the analyte compounds. The use of DI-MS for real time investigations of VOCs has been recently reviewed [3].

Proton transfer reaction—mass spectrometry (PTR-MS) is one of the most established DI-MS methods. It was developed in the early 90s in Prof. Werner Lindinger's laboratory at the Institute of Ion Physics of the University of Innsbruck (Austria). The first PTR-MS instrument was built in 1993 [4,5]. It combined the principles of chemical ionization described by Munson and Field in the 60s [6] with the swarm technique presented by Ferguson and co-workers in the following years [7,8]. The PTR-MS technique has the advantage of allowing real-time monitoring of most VOCs with very low detection limits [9]. Since its first development, PTR-MS evolved as a fast, high sensitivity and non-invasive sensor for volatile compounds and was applied in different fields spanning plant biology, environmental, food science and bio-medicine.

The fundamentals of PTR-MS have been reviewed elsewhere [10,11]. Briefly, in PTR-MS the neutral VOC molecules are ionized via a proton transfer reaction, typically with the hydronium ion (H_3_O^+^). Hydronium ions are generated in a ion source by a hollow cathode discharge on the water vapour which is injected into the ion source region [4]. The purity of H_3_O^+^ ions exiting the ion source usually exceeds 99% [4]. The most significant impurity is constituted by O_2_^+^ ions that are produced either directly by electron impact or by charge transfer from H_2_O^+^ ions. Ions produced in the ion source are allowed to enter into the PTR-MS drift tube. In this region the gaseous samples are also injected. H_3_O^+^ ions undergo proton transfer reaction with most organic species, while such reaction does not occur with clean air constituents. This is because the proton affinity of water is smaller than most VOCs but larger than the constituents of clean air [5]. Proton transfer reactions take place in a buffer gas, usually air, which flows in the drift tube. Protons transfer from primary H_3_O^+^ ions to trace VOCs that have smaller proton affinity than water. The reaction reads:

(1) $${\text{H}3\text{O}}^{+}+\text{VOC}\to {\text{H}}_{2}\text{O}+\text{VOC}\cdot {\text{H}}^{+}$$

At end of the drift tube a mass analyzer detects the ionic products. Proton transfer reaction belongs to the so called “soft ionization” methods, since it is included in the broader family of chemical ionization reactions. In general, soft ionization methods have the advantage of leading to a very limited fragmentation of the product ion, due to the reduced excess energy of the reaction. Hence spectrum interpretation and compound quantification are far simpler than in the case of electron impact. PTR-MS instruments were originally equipped with a quadrupole mass analyser [5]. This has the limit of the low analytical information provided, since the mass resolution is limited to the nominal mass. Recently PTR-MS has been coupled with other detectors, such as ion trap [12,13] and time-of-flight mass analysers [9,14]. As far as ion trap is concerned, there are several advantages compared to the quadrupole mass analyser.

For example, an improved sensitivity is reached with the ion trap for simultaneous investigations of many compounds from a complex gas mixture. In fact in an ion trap all ions are accumulated simultaneously and losses are limited (around 10%) [11], whereas in the case of a quadrupole only a single mass value is analysed at any instant of time.

Time-of-flight (ToF) mass analysers build upon the observation that heavier ions fly more slowly than lighter ions having the same kinetic energy. The great advantage of ToF analysers is the enhanced analytical information provided. In fact they may reach a resolution up to about 7,000 (m/Δm), thus allowing to separate many isobaric compounds. If proper mass calibration is applied to ToF spectra it is also possible to identify the compound sum formula [14]. Beside mass resolution, ToF detectors provide better time resolution and mass range than quadrupoles [9].

Recently, PTR-MS instruments have been coupled with systems that allow the switching from H_3_O^+^ to, for instance, NO^+^, O2^+^, Kr^+^ and Xe^+^ [9,15]. This opens the possibility to detect important compounds such as CO, CO_2_, N_2_O, SO_2_ or ethylene and acetylene, that are not detectable using H_3_O^+^. Moreover the different ionisation induced by different precursor ions allows, in some cases, the separation of isobaric compounds [15–17].

Notably, Italy has been involved in the application of PTR-MS from the very beginning through the activity of some research groups involved in food science and technology from 1999 [18], environmental science from 2001 [19]. Health science, on the contrary, has been addressed only from 2012 [20,21].

In this review we will discuss, on the basis of available literature, the different studies carried on in laboratories based in Italy. Section 2 is dedicated to discuss original and fundamental developments required to further support the application of PTR-MS, such as ion-molecule reaction, chemistry of volatile compounds and data analysis. The subsequent sections are organized according to the different applications and research fields: food science and technology (Section 3), on-line VOC monitoring (Section 4), environmental sciences (Section 5), health and medical sciences (Section 6).

## 2. Italian Contributions to Fundamental Aspects of PTR-MS

Being a relatively new technique, several issues regarding fundamentals of the PTR-MS techniques have been recently addressed and often enhanced. For instance, such aspects encompass VOC concentration determination from PTR-MS spectra [22,23], compound identification [24,25] and determination of Henry's law constants [26]. Moreover PTR-ToF-MS, providing larger and more complex spectra compared to its precursor, has opened several new challenges ranging from dead time correction [27] to accurate mass calibration [24] and automatic peak extraction [28].

### 2.1. Absolute VOC Concentration Determination

In principle with PTR-MS it is possible to absolutely determine VOC concentration without need of calibrating the instrument, provided the compound underlying the signal and the branching ratios are known. Indeed, from Equation 1 it follows that at a first order approximation, VOC concentration is retrieved from the ion signals measured in the mass spectrometer via the following equation [5]:

(2) $$[VOC]=\frac{1}{k\tau }\frac{\left[VOCH^{+}\right]}{\left[H_{3}O^{+}\right]}$$

where *k* is the rate coefficient of the proton transfer reaction governing Equation (1), *τ* is the residence time of the ion in the drift tube, [VOCH^+^] and [H_3_O^+^] are the ion concentrations.

Equation (2) is suitable for VOC concentration estimations when the effect of water cluster ions is negligible [23]. Under high humidity conditions and low *E*/*N* (where *E* is the electric field in the drift tube and *N* is the gas number density) values [29], the amount of water clusters ions present within the drift tube may be substantial and even exceed that H_3_O^+^ of ions, thus a non-negligible rate of proton transfer or ligand switching reactions with VOC may take place [30–32]. Moreover, the residence time may not be well defined [30,33]. Under such conditions Equation (2) is therefore not suitable. The effect of water clusters on rate coefficients *k* and the validity of Equation (2) for a wide range of humidities has been investigated [23]. While no effects are present at dry conditions, for high humidity values the effect strongly depends on *E*/*N* [29]. *E*/*N* values exceeding 120 *Td* strongly limit the amount of cluster ions even at 100% relative sample humidities and therefore Equation (2) still provides a fair approximation [23].

For a rough VOC concentration estimation a constant *k* = 2 × 10^−9^ cm^3^/s is sometimes plugged into Equation (2). This introduces a systematic error that is usually below 30% for most compounds [22,23]. In many applications values of k determined under thermal conditions in the drift tube are employed. Such a practice is questionable [11,22], since collisions between ions and neutral molecules in the drift tube occur at much higher energy than just the thermal one. For instance, typically the centre-of-mass energy of the ion-molecule system is about 0.2 eV while the thermal energy is about 2.25 meV. This is not problematic for non-polar molecules since the additional energy does not change their proton transfer reaction rate coefficient *k* [22]. At the contrary, for polar molecules the thermal *k* differs from the actual *k* and the error can exceed 30% for molecules with high dipole moment. Therefore it is recommended to employ rate coefficients estimated at the correct energetic conditions. Various estimation approaches are possible [22]. A full list of compounds with the corresponding *k* estimated at various typical PTR-MS energetic conditions is provided in the supplementary materials of [23], which are freely accessible on-line.

It is also possible to experimentally determine rate coefficients via PTR-ToF-MS [23]. Agreement within a few percents with theoretical values is found. Values of *τ* for a standard PTR-MS are close to 100 μs. A proper determination of *τ* may be carried out experimentally [30] or theoretically [23] by:

(3) $$\tau =\frac{l^{2}}{\mu U}$$

where *l* is the length of drift tube (typically 9.3–9.8 cm for Ionicon instruments), *μ* is the mobility of the H_3_O^+^ ion in the drift tube (experimental values can be found for example in [30]) and U is the electric potential applied to the drift tube (typically in the range 400–600 V).

The [VOCH^+^]/[H_3_O^+^] ratio cannot be straightforwardly determined using the ion count rates in the mass spectrometer. In fact there is an important caveat. The different detection efficiencies of the mass spectrometer for ions having different masses must be taken into account. In the case of PTR-MS equipped with a quadrupole mass analyser, mass discrimination can be experimentally determined following the procedure described in [34].

**Figure 1.**
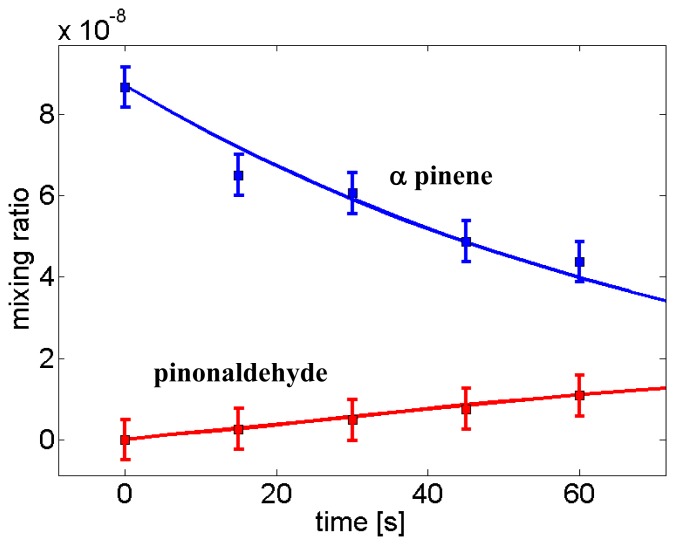
Absolute VOC concentration determination with PTR-TOF-MS. Predicted and measured decrease of alpha-pinene concentrations in a flowtube along with the production of the dominant first order generation product pinonaldehyde upon exposure to ozone. Comparison between model prediction (solid lines) of mixing ratios for alpha-pinene (blue) and pinonaldehyde (red) and measured values by PTR-ToF-MS using Equation 4 (squares) showing an agreement within 10%. Reprinted with permission from ref [23]. Copyright 2012 American Chemical Society.

For PTR-ToF-MS a very simple theoretical approach is also possible [23], leading to transforming Equation (2) into:

(4) $$[VOC]=\frac{1}{k\tau }\frac{{\left[VOC\cdot H^{+}\right]}_{\text{measured}}}{{\left[H_{3}O^{+}\right]}_{\text{measured}}}\frac{\sqrt{{\left(m/z\right)}_{H_{3}O^{+}}}}{\sqrt{{\left(m/z\right)}_{VOC\cdot H^{+}}}}$$

where now [VOCH^+^]_measured_ and [H_3_O^+^]_measureed_ are the ion count rates in the mass spectrometer. [H_3_O^+^]_measureed_ can be estimated from the signal of the isotope of H_3_O^+^ at *m*/*z* = 21.0221 Th. In this case $\sqrt{{\left(m/z\right)}_{H_{3}O^{+}}}$ must be replaced by $\sqrt{21.0221}$. Since fragmentation of the protonated VOC may occur, in order to properly estimate VOC concentrations branching ratios must be employed or equivalently Equation (2) or (4) must be used for all fragments separately and their results subsequently summed up. This approach leads to a quantitative determination of VOC concentrations by PTR-ToF-MS (Figure 1) [23].

### 2.2. Compound Identification

Compound identification in PTR-MS is challenging and usually requires further information to be pursuable. Having unit mass resolution, PTR-Quad-MS instruments only provide nominal mass determination and several compounds may be superimposed on the same peak. While this is not a problem if PTR-MS is employed to have a fingerprint of the sample or to monitor rapid changes of known compounds, it is certainly a limitation for the identification of compounds of unknown identity. Corroborating tentative identifications with, e.g., compound identification by GC-MS, is often the most straightforward choice. In specific applications, extra information beside the nominal mass may be gathered by considering desorption kinetics [35]. Specific classes of compounds display different desorption behaviours. For example “sticky” compounds such as carboxylic acids are characterized by desorption curves that decrease much more slowly than other classes of compounds such as, for instance, esters. Such observations are in certain circumstances useful to restrict the number of candidate compounds [35].

Given the higher mass resolution, PTR-ToF-MS strongly enhances compound identification. Although isomers are not distinguishable, the compound sum formula is in principle determinable. It is crucial to achieve a good mass accuracy in the spectra. In fact in many cases a mass accuracy of 5 ppm is sufficient for sum formula determination. The acceptable accuracy depends on the application and on the *a priori* knowledge of the measured samples. For instance, suppose that an unknown peak at *m*/*z* = 205.1951 is measured in the headspace of a food sample [24]. Given the considered matrix, it may be reasonable to restrict the choice to ions encompassing only C_0–100_, H_0–100_, N_0–20_, O_0–20_ and S_0–20_. Within an uncertainty of 15 ppm, there exists only one possible structural sum formula.

PTR-ToF-MS coupled with suitable internal calibration enables to reach a mass accuracy as low as less than 5 ppm for a wide range a *m*/*z* values [24]. It must also be pointed out that the actual mass accuracy may depend on the complexity of the peak structure and on the peak extraction procedure [28,36]. If the peak under investigation is inserted in a peak structure involving many other neighbour peaks having close *m*/*z*, then an important role is played by the performance of the analysis procedure in disentangling such structure.

Provided the sum formula has been identified, the step towards compound identification might not be trivial. Fragmentation, complex peak structure and/or the presence of isomeric compounds may still render the challenge unpractical, especially in complex matrices.

### 2.3. Spectra Analysis in PTR-ToF-MS

Spectra produced by a PTR-Quad-MS are relatively simple, being composed by a few hundred numbers that can directly feed further data analysis procedures. At the contrary, PTR-ToF-MS spectra are typically 1,000 times larger [9], involving hundreds of thousands of numbers. The complexity of the spectra is also much increased. Peak extraction procedures are mandatory in order to extract manageable datasets which can be used as inputs for data visualization or data mining procedures. Other issues encompass mass calibration [24] and detector dead time effects [27].

As already pointed out, mass accuracy is of fundamental importance in PTR-ToF-MS for compound sum formula identification. Furthermore, achieving good accuracies is mandatory for applications requiring to average over several spectra, otherwise the resulting average spectrum could be disrupted. Mass calibration in PTR-ToF-MS raw data is nowadays limited to external calibration which implies fixing a set of calibration coefficients employed during the entire acquisition. However because of fluctuations in instrumental parameters, such procedure does not guarantee good accuracy for a sufficient long time, hence the need for internal calibration. The mass calibration procedure depends on the formula used to link the ion time-of-flight with the ion mass. In force of theoretical considerations [37], in principles a two parameter function should be sufficient [24]. However, heuristic investigations showed that better accuracy is provided by a second order polynomial function involving three parameters when Gaussian functions are used to fit the peak shape [24].

Another issue in PTR-ToF-MS is the instrumental dynamic range. Commercial instruments are equipped with ToF systems whose linearity is affected by the detector dead time [38]. This means that ions reaching the detector during the dead time which follows a previous arrival event are irrevocably lost thus causing distortion of intense signals. Analytical corrections of such effects are commonly based on Poisson statistics and are suitable up to intensities of a few ions/pulse [27]. Limitations are basically caused by an increase in apparent noise with the arrival rate [39]. In several applications it is often possible to overcome this problem by using the signal corresponding to an isotope of the ion that has too high intensity. If this not the case, a calibration method [27] has been proposed to extend the dynamic range of at least one order of magnitude beyond the range of applicability of corrections based on Poisson statistics.

Recently the first complete methodology has been proposed to automate the analysis workflow from PTR-ToF-MS raw spectra to data mining [28]. It starts with the already discussed dead time correction and internal calibration followed by baseline removal, denoising, peak detection and intensity extraction. The output is a data matrix of peak intensities, analogous to PTR-Quad-MS data matrices. Preliminary data visualization methods or advanced data mining procedures may be then straightforwardly applied.

### 2.4. Data Mining, Multivariate Analysis and MS-e-Nose

The first applications by Italian scientists in food science and technology [40] indicated the possibility to use the PTR-MS spectra as fingerprints to rapidly identify food samples, that is, to use PTR-MS as an MS-e-nose, a particular implementation of an electronic nose where the array of solid state sensors is replaced by a mass spectrometer. The use of PTR-MS seems particularly suited in this case because it preserves a lot of analytical information (concentration estimation and little fragmentation) in comparison with the application of electron impact ionisation typically used in MS-e-noses. The best way to efficiently exploit this spectrometric fingerprint and a “trademark” of Italian applications of PTR-MS in food science, is through unsupervised multivariate methods for data compression and visualisation [41] and through the setting of classification [40,42] or calibration models [43] by supervised multivariate methods and data mining.

Efficient feature selection methods has been studied in the case of PTR-Quad-MS data [44] but they are now even more important in the case of PTR-ToF-MS spectra because of their higher complexity and size [28]. The methods developed over time on the problem of PTR-MS data analysis have been summarised by Cappellin *et al.* [28].

Multivariate analysis is also useful in building multivariate models linking PTR-MS fingerprints with compound concentrations measured by well-established techniques such as GC-MS. LASSO and PLS have been used to predict the intensity of GC-MS peaks from PTR-ToF-MS fingerprints in olive oil and grana cheese matrices [25]. The two multivariate methods generally provided similar prediction performances but LASSO showed better interpretability of the model coefficients.

## 3. Italian PTR-MS Applications in Agrifood Science

Given the importance of VOCs in food flavour and quality assessment, agrifood science is seeing major breakthroughs from the possibility of employing suitable analytical methods for VOC identification, quantification and monitoring. Already in the early days of PTR-MS, Werner Lindinger and his team, who had developed the PTR-MS technique at the Innsbruck University, understood the advantages of PTR-MS for food science and carried out pioneering applications also in collaboration with companies [45]. Nowadays PTR-MS is becoming well-established in agrifood science and in particular it is the reference technique for in-nose direct analysis applications where monitoring in real time at high sensitivity is crucial [46]. This aspect will be discussed in Section 4.

Italian studies are giving major contributions, spanning from food quality assessments to flavour perception and process monitoring. Some ideas, as the linking with sensory analysis or with genomic data, have been successfully exploited. The review of the main results will consider the (a) works based on the application of the PTR-Quad-MS for the semi-static analysis of the head space of food samples also in correlation with sensory analysis and other instrumental characterisations (Section 3.1.1 and Section 3.1.2); (b) more recent results based on the Time of Flight version (Section 3.1.3); (c) the use of PTR-MS as a rapid tool for fruit phenotyping and QTL studies (Section 3.2); (d) works exploiting the high time resolution of the PTR-MS (Nose space analysis and bioprocess monitoring, included in Section 4).

### 3.1. Food Quality Assessment by Headspace Analysis

#### 3.1.1. PTR-Quad-MS for Rapid and Non-Invasive Food Characterisation

The Italian pioneering application of PTR-MS to food quality was conducted by the research group of Salvatore Iannotta at CeFSA (Trento, Italy) in collaboration with the team led by Prof. Lindinger [18]. It dealt with the problem of fruit postharvest management, namely the non-invasive monitoring of the evolution of VOC emission during berry aging after harvest. Strawberries, blackberries, raspberries, blueberries, white and red currants were monitored while aging at room temperature. The considered PTR-MS peaks included those tentatively related to methanol, ethanol, acetaldehyde, acetone, acetic acid, esters and ketones. Specific levels of VOC emission and time evolution behaviours were found for the different berries, indicating possible markers to assess ripening or decaying stage. This study showed the possible advantages of PTR-MS. Typically VOCs are analysed by chromatographic techniques, requiring sample preparation or pre-concentration, while PTR-MS allows a non-destructive analysis by direct injection of headspace air and moreover it is more selective than other techniques such as electronic nose that had been attempted to assess total emission of aromatic VOCs in berry fruits [47].

Food ripening was also the topic of a study by Aprea *et al.* [43], who investigated VOCs in the headspace of young and ripened Trentingrana cheeses. Cheese loaves were collected from all the main cheese-factories belonging to the Trentingrana consortium. Despite the variability between the samples, significant differences in headspace VOCs were detected between young and ripened samples. In particular esters and acetaldehyde were found to markedly increase with ripening. Multivariate modelling via PLS provided good cross-validated predictions of the ripening age from the PTR-MS fingerprint.

Assessments of food quality by PTR-MS also comprise rapid detection of food spoilage. Aprea *et al.* [48] characterized the headspace of virgin olive oil samples from Tuscany comparing control and defective samples. Sample spoilage by oxidative processes was assessed by a panel of trained judges that indicated the presence of very moderate ‘rancid’ flavour in the spoiled samples. PTR-MS coupled to multivariate classification via LDA on compressed data by dPLS provided 100% correct classification of the samples, thus indicating the viability of an automatic approach for oxidative alteration detection in olive oil. Spoiled samples were characterized by an increase in aldehydes such as heptanal, octanal, nonanal, which were also positively correlated to the peroxide value. Other compounds not correlating with peroxide were also found to differ between control and ‘rancid’ samples. Indeed, heptanal concentration was higher in spoiled oils while hexenal was lower. Aprea *et al.* also monitored on-line oxidative alterations in headspace VOCs of olive oils during thermal treatments, following the time-evolution of several aldehydes [48,49].

The characterization of the effect of food treatment by PTR-MS was the scope of Biasioli *et al.* [50]. Red orange juices from the same industrial batch were processed with four different treatments: untreated, standard-pasteurisation, flash-pasteurisation and high-pressure stabilisation. The results of PCA and ANOVA reflected a marked separation between the heated samples and the other ones. This work highlighted for the first time the possible use of PTR-MS as an MS-e-nose (fingerprinting mass spectrometry) and the importance of coupling PTR-MS with automatic data analysis. In comparison with other MS-e-nose based methods, PTR-MS has the advantage to provide richer analytical information [3].

Gasperi *et al.* [51] explored by PTR-MS and other methods the effects of two innovative techniques to treat apple juice, namely supercritical CO_2_ treatments and N_2_O pasteurization. No differences between control and treated samples were found in the chemical analysis of soluble compounds and only moderate effects on the food quality were perceivable by a sensory panel of trained judges. On the contrary, PTR-MS analysis detected a dramatic depletion of VOCs in the juice headspace of the treated samples. Real time monitoring of the treating process showed the depletion of about one third of the total measured headspace concentration during the first 10 min of treatments.

Aprea *et al.* [52] measured headspace VOCs in the raspberry cultivars ‘Polka’ and ‘Tulameen’ by both PTR-MS and SPME/GC-MS. Fresh (whole and smashed) fruits and juices were considered. In all cases the volatile emission profiles of the two cultivars were measured to be significantly different by both headspace techniques, with ‘Tulameen’ displaying a higher concentration of aldehydes and alcohols, in particular hexanol and hexanal which confer notes of herbaceous flavour and odour. Juice were also found to have different profiles from smashed fresh fruits, displaying *e.g.*, lower concentration of esters and higher concentration of alcohols and aldehydes. This work also highlighted the suitability of PTR-MS in measuring some VOCs, such as methanol and acetaldehyde, which are not easily measurable with SPME/GC-MS. Within the same study relevant PTR-MS data on the real time evolution of VOC emission during the smashing of raspberry fruits are also presented. Typical wounding products, such as C6-VOCs emitted by fruits, were found to dramatically increase after smashing.

Headspace VOC fingerprint by PTR-MS provides a potential tool for discriminating not only treated and control samples but also different varieties of the same food type. Nine cultivars of strawberries from different locations were studied over three years. By only employing PTR-MS fingerprints of the strawberry samples and advanced data mining methods, successful models to classify the different cultivars were developed [40,42]. The most relevant PTR-MS peaks in the cultivar discrimination problem were also investigated using RF-RFE and SVM-RFE, showing that a reduced set of peaks that are highly relevant to the problem may be identified. These works indicated a methodology that has been applied in different case studies, and in particular to the classification of fruit cultivars, which will be also discussed below in the Section 3.1.3 [53,54].

Beside the importance for quality control, these studies also demonstrate the potential of PTR-MS in supporting and enhancing fruit breeding programs by allowing rapid and non-destructive characterizations of fruits to be selected.

PTR-MS has also been applied to enology, differentiating the headspace VOC fingerprints of wines produced from different grape varieties, after dilution by a factor of 1:40 with pure nitrogen in order to prevent interferences from ethanol in the measured ions [55].

The discrimination potential of PTR-MS may be not only important in cultivar characterization but also in product origin separation. Preliminary indications in this sense are present in [56]. The headspace of white truffles (*T. Magnatum pico*) from six different origins in Italy was studied with both PTR-MS and SPME/GC-MS [56]. Partial discrimination of product origin by PTR-MS fingerprint was shown, nonetheless definitive conclusions could not been drawn due to the small number of investigated samples. Several compounds were tentatively identified in PTR-MS spectra, using fragmentation patterns and GC-MS data in support. Sulphur containing VOCs dominated the spectra, in particular dimethyl sulphide and bis(methylthio)methane. A high positive correlation between GC-MS signals and single PTR-MS peaks were also found for several compounds, including dimethyl sulphide, dimethyldisulphide, bis(methylthio)methane. The comparison of the two analytical techniques for white truffle headspace analysis allowed to point out that PTR-MS provides a “more realistic distribution of the single molecules quantified through PTR-MS without pre-concentration” [56]. The activity of Saskia van Ruth and co-workers demonstrates the pros and cons of this approach to food traceability and origin control [57].

#### 3.1.2. Linking PTR-MS with Sensory Analysis

A second original idea proposed by Italian scientists was the linking of the fast PTR-MS fingerprinting with other characterisation methods of food samples and, in particular, with sensory analysis. Being a time consuming and expensive approach, any method that can reduce the application of sensory analysis to a preliminary calibration phase is relevant for the agroindustry.

Italian contributions employing PTR-MS in this context mainly concentrate on dairy products. The first study was carried out considering seven different brands of mozzarella cheese [41]. Both sensory evaluation by a train panel of 8 judges and chemical analysis of food aroma by PTR-MS were performed. Indications of similar class discrimination capability by the two approaches were found upon comparison of PCA results for the two datasets.

Grana cheeses have been the subject of other studies [58,59]. Boscaini *et al.* [58] showed that a similar discrimination between Grana Padano, Parmigiano Reggiano and Grana Trentino cheeses is achieved by both PTR-MS headspace analysis and gas chromatography-olfactometry.

A multivariate modelling of the link between sensory evaluations by a trained panel and PTR-MS measurements was carried out for 20 Trentingrana cheeses [59]. In the case of several odour and flavour attributes reasonable prediction performances were achieved using partial least squares as calibration method.

Besides its importance in aroma profiling, sensory analysis can also be used to estimate outdoor and indoor air quality and in particular odour emissions from industrial plants. This aspect will be exploited in Section 5.3 where the relation between PTR-MS and olfactometry will also be discussed.

#### 3.1.3. PTR-ToF-MS in Food Science and Technology

A major breakthrough for PTR-MS applications has been the replacement of quadrupoles, used in the first generation of instruments, by Time-of-Flight mass analysers [9], which provide higher mass and time resolution and a wider mass range. This allows the monitoring of very fast processes up to 10 Hz, the separation of molecules with the same nominal mass and, in most cases, the association of a unique sum formula to the spectrometric peaks.

The very first (Italian and worldwide) published application of PTR-ToF-MS to food quality assessment was devoted to study the effect of milk storage conditions on the headspace VOC profile of Trentingrana cheese [60]. Four milk storage procedures on both summer and winter productions of one cheese factory belonging to the Trentingrana consortium were tested. Given the high amount and complexity of the data, partially automated data analysis procedures were employed [24,28]. The achieved mass accuracy allowed determining the ion sum formula for most peaks.

**Figure 2.**
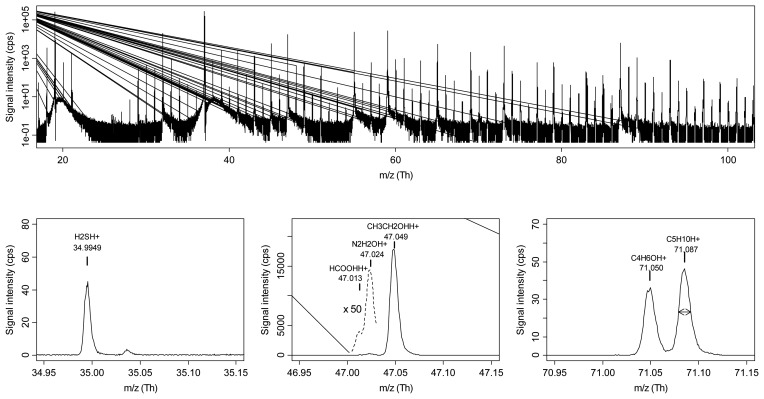
PTR-TOF-MS spectrum of a cheese sample headspace. The upper panel shows the mass region (20–100 Th). The lower panels enlarge the regions around few selected peaks. Reprinted with permission from ref. [60]. Copyright 2010 John Wiley & Sons, Ltd.

Moreover, especially for low *m*/*z* values, in many cases compound identification was possible, by taking also into account fragmentation patterns of pure standards. Peak identification was further confirmed by SPME/GC-MS analysis. Advanced data mining methods allowed obtaining a good discrimination between the sample classes for both summer and winter production. Furthermore, RE-RFE provided a straightforward way to identify the most relevant peaks in the discrimination. Esters were found to play a major role in the classification of samples belonging to the summer production, while aldehydes and ketones highly contributed to the discrimination of winter grana cheeses. Also sulphur and nitrogen-containing VOCs, such as dimethyl disulphide and dimethylpyrazine, showed differences among the classes. The study well highlighted the advantages of PTR-ToF-MS compared to its precursor PTR-Quad-MS. Since separation of isobaric compounds is achieved with PTR-ToF-MS, the simultaneous monitoring of multiple peaks at the same nominal mass, even at very different concentrations, is possible. For instance ethanol and formic acid are separated (Figure 2), as well as other peaks that were interfering in PTR-Quad-MS spectra, such as methanol and the isotope of oxygen at nominal mass *m*/*z* = 33. The presence of trace contaminant, *e.g.*, acetonitrile in the case of some cheese samples, is also easily detected in spectral regions where this could not be possible with PTR-Quad-MS.

The effectiveness of PTR-ToF-MS to study treatment effects on complex food matrices has also been shown by other studies. Soukoulis *et al.* [61] investigated the effect of matrix composition on the yogurt headspace concentration of endogenous VOCs as well as on physicochemical and textural properties. Samples with various concentrations of milk fat, solid non-fat and modified tapioca starch were analysed, using a PTR-ToF-MS for VOC concentration determination. Low fat systems showed a higher concentration of acetaldehyde, 2-propanone, 2,3-butnedione, 2,3-pentanedione and 2-butanone, while lower concentrations of ethanol and acetoin were found. The addition of modified tapioca starch only enhanced ethanol emission. The effect of stirring was also investigated, highlighting that gel breakdown due to yogurt stirring only affected ethanol, acetoin and 2,3-penatnedione emission, which were significantly lower in set yogurts [61].

**Figure 3.**
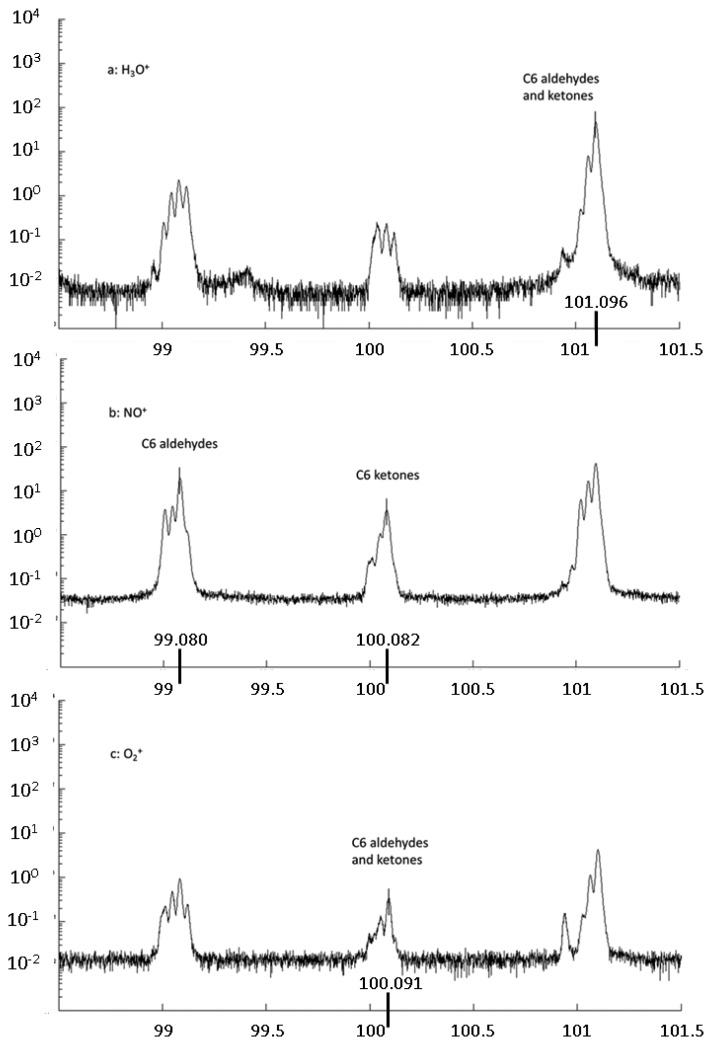
Dry cured ham headspace. Exemplificative peaks related to aldehydes and ketones measured by PTR-ToF-MS using different reagent ions. a: H_3_O^+^; b: NO^+^; c: O_2_^+^. Reprinted with permission from reference [63]. Copyright 2012 Elsevier Ltd.

Sánchez del Pulgar *et al.* [62] characterized dry-cured hams differing in origin (Iberian and Italian) and in processing conditions. Four different Protected Designations of Origin (PDO), namely the Iberian Dehesa de Extremadura and the Italian Prosciutto di Parma, Prosciutto di San Daniele and Prosciutto Toscano, were considered. Data mining methods, in particular PDA, provided 100% sample classification based on fast headspace VOC analysis by PTR-ToF-MS. Iberian dry-cured hams displayed higher overall concentration of VOCs compared to Italian ones. Sulphur compounds such as dimethyl sulphide and methanethiol differed between the two origins. Moreover compounds probably linked to the processing conditions (e.g., treatment nitrates and nitrites) were identified, such as for instance acetonitrile and hexanenitrile. In general, peaks corresponding to aldehydes and ketones were higher in Iberian dry-cured hams. However, the primary ion used in the study (H_3_O^+^) did not allow achieving separation of the two compound classes. Therefore, in a later work Sánchez del Pulgar *et al.* [63] employed NO^+^ as primary ion as well as H_3_O^+^ and O_2_^+^. In this pioneering application of CTR-ToF-MS to complement results from PTR-ToF-MS, it is shown that NO^+^ as primary ion leads separate aldehydes and ketones (Figure 3), thanks to the type of ionization reactions that the two classes of compounds undergo with NO^+^. The study focused on the discrimination of Iberian dry-cured hams based on the rearing system, on the one hand pigs fattened on pasture and acorn (Montanera); on the other hand pigs fattened on high-oleic concentrated feed (Campo). A good discrimination based on headspace VOCs of both lean and subcutaneous fat was achieved upon data mining by PDA. At last, it was also studied the effect of the insulin-like growth factor II (IGF-II) polymorphism—a QTN involved on fat deposition in pigs—on the final quality characteristics of Iberian dry-cured ham [64]. A negligible effect of the IGF-II on the VOC profile as detected by PTR-ToF-MS was found.

**Figure 4.**
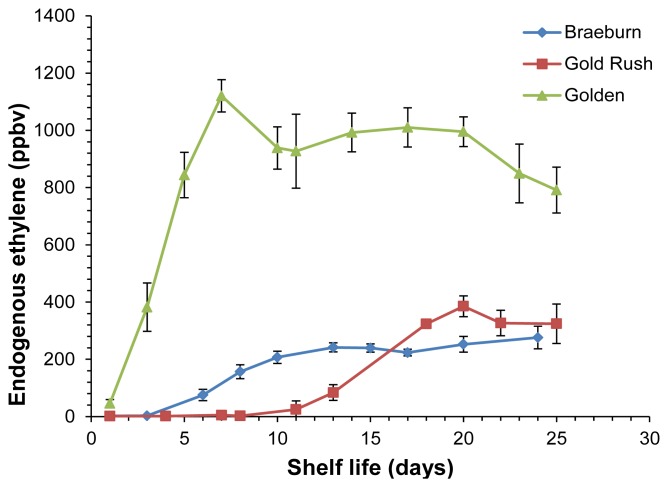
Evolution of endogenous ethylene emission for Breaburn, Gold Rush and Golden Delicious apples during shelf life at 20 °C under ambient air room conditions. Reprinted with permission from [65]. Copyright 2012 Springer Science+Business Media, LLC.

A different application addresses a technical post-harvest problem, namely the VOC evolution monitoring during post-harvest shelf life ripening of fruits. Soukoulis *et al.* [65] studied the headspace of three well known apple cultivars (‘Golden Delicious’, ‘Gold Rush’ and ‘Breaburn’) during 25 days after harvesting. Remarkably, PTR-ToF-MS provided evolution curves not only for ions corresponding to protonated VOCs but also of C_2_H_4_^+^, associated to ethylene. Having lower proton affinity than water, ethylene does not undergo proton transfer from H_3_O^+^ ions and thus should not be detected. However, residual O_2_^+^ from the ion source efficiently reacts with ethylene via charge transfer thus producing C_2_H_4_^+^ ions. The possibility to monitor ethylene evolution is of outmost importance in apple post-harvest management, since this hormone is strongly involved in the regulation of aroma production [66], especially for specific VOC groups such as esters and alcohols [67]. Ethylene evolution showed a clear pattern, displaying an initial increase followed by a gradual decline, as already generally observed in the literature (e.g., [68]), with ‘Golden Delicious’ being characterized by higher ethylene emission than ‘Gold Rush’ and ‘Breaburn’ (Figure 4). Esters and α-farnesene evolution during post-harvest showed a close connection to ethylene emission, while carbonyl compounds presented a different time evolution and methanol and ethanol remained barely constant for all investigated apple cultivars. A systematic study on the possibility to quantify ethylene by PTR-MS is currently ongoing in our laboratories.

**Figure 5.**
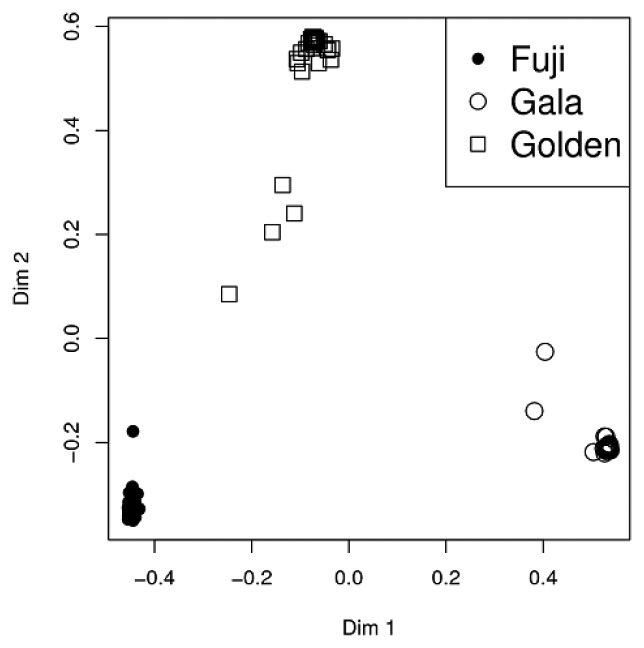
Headspace VOC analysis of apple clones with PTR-ToF-MS. Random Forest graphical output for the discriminant analysis of the PTR-ToF-MS data of apple clone samples. Reprinted with permission from [54]. Copyright 2012 Springer Science+Business Media, LLC.

PTR-ToF-MS coupled to suitable data mining methods has a great potential for investigation in the field of metabolomics. In fact fruit cultivars and even clones may possibly be discriminated in relation to important secondary metabolites, such as volatile organic compounds. Cappellin *et al.* [54] investigated headspace VOCs in 15 different clones of 3 well known cultivars (‘Gala’, ‘Golden Delicious’ and ‘Fuji’). All samples could be correctly classified by RF in relation to the corresponding cultivar class (Figure 5). Moreover some of the clones could be discriminated and in the case of ‘Gala’ clones estragole and hexyl-2-methylbutanoate were also identified as markers for the separation. The achieved mass accuracy allowed for sum formula determination of the peak of interest, while compound identification was based on supporting SPME/GC-MS measurements on selected samples. The study pioneered the possibility to characterize fruit clones on the basis of their VOC emission profiles, opening new horizons in view of potential applications in breeding programs and royalty management.

### 3.2. Genetics and Fruit Phenotyping by PTR-MS

Aroma is one of main factors considered in the modern definition of fruit quality, as well as texture, appearance and nutritional properties [69], strongly impacting consumer appreciation. However, the analysis of the aroma trait in a broad number of samples may be laborious and time consuming. The study of the link between fruit genotypes and aroma profile has therefore attracted increasing attention in recent years. A valuable tool in selection programs are molecular markers associated to the aroma trait that can be employed in assisted breeding programs. A first step in this direction is the mapping of Quantitative Trait Loci (QTLs), which is the identification of DNA regions which are associated to quantitative traits such as aroma. QTL investigations require characterizing the aroma profile of a large number of fruits, thus fast techniques such as PTR-MS are particularly suited for this application.

The first study in this direction was carried out by Zini *et al.* [70], who considered two populations of 86 apples belonging to the progeny ‘Fiesta’ × ‘Discovery’. Ten genomic regions associated with seven PTR-MS peaks were identified. In particular, QTL related to the emission of esters were found in linkage group 2. Moreover, a highly significant QTL was determined in association to *m*/*z* = 28, putatively associated to the hormone ethylene. These results have been confirmed with larger populations harvested in three different locations [71].

Another important crop, *i.e.*, strawberry, was investigated by Carbone *et al.* [72] in order to elucidate the connection between gene expressions and aroma traits as described by PTR-MS measurements. The study confirmed the possibility to distinguish strawberry genotypes by means of PTR-MS analysis using data related to three locations, three harvesting times and different storage conditions and duration. A positive correlation was found between the expression of an AAT gene and ester emission.

The application of PTR-MS as powerful phenotyping tools is at the very beginning and up to now has only been exploited by Italian groups. Still unexplored is the even greater potential of the ToF version of PTR-MS, that, by providing richer analytical information, may dramatically increase the number and quality of phenotypic traits that it allows to simultaneously investigate. Therefore new studies in this direction are expected in the near future.

## 4. On-Line Monitoring: When High Time Resolution Matters

Because of its high time resolution coupled with high sensitivity, PTR-MS is one of the best “sensors” to be used in process monitoring. The Italian PTR-MS community has been active also in this context: some example has been already mentioned above as wound compound emission from plants, the real time monitoring of VOC emission from olive oils subjected to an oxidative process induced by heating exposure [48,49], the monitoring of headspace VOCs during raspberry fruit smashing [52] and the monitoring for benzene formation from benzoate in model systems by proton transfer reaction-mass spectrometry [73]. Here we manly concentrate on two emblematic examples as bio-process monitoring and nose-space analysis.

### 4.1. Bioprocessing

After the commercialization of PTR-ToF-MS in 2008 [9], the first report of its application to dynamic biochemical process was published in 2010 [74]: Soukoulis *et al.* monitored lactic acid fermentation, an important dynamic bioprocess of relevance to the dairy industry, being involved in the production of many dairy products such as fresh cheeses, yogurt, acidified diary beverages and desserts. This study is important in view of possible industrial applications for quality control of the final product or detection of inefficiencies in process operations highlighted by VOC evolution [75]. Moreover VOC monitoring could complement or even outplace pH monitoring, which is the common practice in the dairy industry but displays several drawbacks [76]. Other groups attempted the use of electronic nose to continuously monitoring lactic acid fermentation but the poor selectivity of the instrument limited the distinction of the emitted VOCs. On the contrary Soukoulis *et al.* employing a PTR-ToF-MS simultaneously followed the emission of several specific VOCs, including VOCs of major importance such as acetaldehyde, acetoin, diacetyl, propanone; VOCs impacting flavour formation; off-flavours such as DMS, furfural, and methanethiol. Compound identification was supported by off-line SPME/GC-MS analysis of inoculated milk and final product after fermentation occurred. The possibility to monitor off-flavours provides a potential tool to indicate malpractices in manufacturing procedure. Clear trends were observed in the evolution of some compounds, possibly reflecting biochemical activities and effects on flavour release of the gel matrix developed during fermentation. The applicative relevance of this approach is demonstrated in follow up studies where PTR-ToF-MS allowed the characterisation of many samples and the monitoring of relatively fast kinetics which would have been hardly affordable by gas-chromatographic analysis.

Biofuel production and waste bioprocessing are further applications where a rapid and non-invasive sensor can be very useful. In this sense PTR-ToF-MS has also been exploited for bioprocess control during biogas production [77]. Biogas represents a valuable renewable fuel in alternative to fossil ones. Nowadays one of its most promising applications is in Solid Oxide Fuel Cells [78]. One way of producing biogas is offered by Organic Fraction of Municipal Solid Waste through anaerobic digestion [79]. During the process mainly methane and carbon dioxide are produces but also relevant fractions of VOCs, some of which could have potential detrimental effects in subsequent operations [80]. Papurello *et al.* [77] monitored digestion processes for 30 days measuring methane (by an infrared detector) and carbon dioxide (by an electrochemical cell) as well as VOCs by PTR-ToF-MS. Tentative identification of tens of VOCs was based on sum formulas determined from ToF spectra and was supported by SPME/GC-MS analysis. Monitored VOCs included sulphur compounds, ammonia, alcohols, carbonyl compounds, carboxylic acids, terpenes and aromatic compounds. Most of them exhibited a well-defined emission pattern reproducible on independent experiments and characterized by a double-peak emission which probably reflected the combined effect of volatilized or oxidized biomass organic compounds and microbial degradation of organic substrates.

### 4.2. Nose-Space Analysis

In order to obtain a complete picture in food perception, it is not sufficient to analytically characterize the flavour of food and to carry out evaluations by sensory judges. Aroma does not only depend on food VOC composition but also on the way it is perceived by the olfactory system. What actually directly interacts with the olfactory receptors may not be the same VOC profile that is measured on the food headspace. It is therefore important to monitor the flavour release “*in vivo*” during food consumption. In practice, the VOCs present in the air exhaled through the nostrils are profiled. This is the so called “nose-space” measurement [81]. PTR-MS is particularly suited in this field providing on-line monitoring of nose-space at typically better sensitivities than alternative techniques [46]. Moreover the measured compounds are often known and hence the lack of straightforward compound identification is not a dramatic limit.

Italian contributions in nose-space studies by PTR-MS are also emerging in recent years. Aprea *et al.* studied flavour release from 21 subjects consuming flavoured custards with different textures [82]. The nose-space concentration of flavour compounds including esters and acetaldehyde, as well as the concentration of breath related compounds such as acetone, were continuously monitored by PTR-MS. It emerged a marked inter-individual variability and the oral processing protocol notably influenced the flavour concentrations. Different effects of the custard texture on nose-space VOC concentrations were observed among the panellists.

Ting *et al.* [83] carried out in-nose measurements of acetaldehyde, ethanol and selected esters during consumption of six common apple cultivars. Despite the inter-subject variability, marked correlations between textural properties and parameters describing the time curves of flavour release were found. Moreover substantial differences among the investigated cultivars were observed in the nose-space VOC concentration patterns. In general it was pointed out that in-nose release of aroma compounds is a multi-factorial process, affected by both exogenous (e.g., cultivar, subject) and endogenous drivers (e.g., oral physiology).

Recently the first application of PTR-ToF-MS has been performed in the laboratories of Fondazione Edmund Mach [84]. Both *in vitro* and *in vivo* measurements of flavour release have been carried out using the same instruments. Six cereal bars with varying sugar compositions were investigated, finding that the nose-space concentrations of important compounds including acetaldehyde, alkyl esters and carboxylic acids are affected by the sugar composition. At the contrary no effect was observed for methyl cinnamate.

The advantages of employing PTR-ToF-MS instead of its precursor PTR-Quad-MS for nose-space analysis are at least two-fold. On the one hand, with a quadrupole detector only a few masses can be measured at the sampling rate required to resolve breath patterns. In fact, typically in order to measure a single *m*/*z* channel about 200 ms are needed as a trade-off between signal to noise ratio and sampling time. In a similar time span, the full mass spectrum can be acquired using PTR-ToF-MS. On the other hand the compound nominal mass information provided by PTR-Quad-MS is often not sufficient for unambiguous compound identification because of isobaric compounds and fragmentations, especially when complex mixtures are analysed.

### 4.3. Estimations of Henry's Law Constants

PTR-MS allows to experimentally estimate Henry's law constants via a gas stripping procedure [85]. Aprea *et al.* [86] reported measurements of water-solutions/air partition coefficients and ethanol-solution/air partition coefficients for methyl and ethyl esters using such technique. For water solutions they estimated Henry's law constants that are systematically lower than those reported in the literature using GC-MS. GC-MS suffers from adsorption of VOCs on walls, tubing or injection system that may lead to overestimate Henry's law constants [87], while this is not the case for PTR-MS [85].

Schuhfried *et al.* [26] used the same technique to measure Henry's law constants for selected monosulphides and disulphides. The results were consistent with those reported by Pollien *et al.* [88] using a similar approach but were consistently smaller of about 10% than experimental literature data based on GC-HS measurements [26], confirming the findings of other works [85,86]. Available and freely accessible models for Henry's law constant estimation were also tested and compared to the newly available experimental data. The tested models (Henrywin, LSER, SPARC) were affected by the drawback of the scarcity of reliable literature data and required adjustments [26].

### 4.4. Characterisation of Fast Stimuli for Insect Electrophysiology

In insect electrophysiology, fast bursts of volatile compounds diluted in air are used to stimulate insect antennas. Their proper characterisation (both time profile and intensity) is mandatory but difficult to achieve. Spectroscopic methods are often used but they lack of specificity and sensitivity. Recently, Tasin *et al.* [89] demonstrated how PTR-ToF-MS can measure the fast transients related to insect electrophysiology and estimate concentrations for almost thousand samples. Unlike spectrometric methods, PTR-MS allows for the separation of possible contaminants.

## 5. Italian PTR-MS Applications in Environmental Science

PTR-MS technology has wide applications in environmental sciences. In Italy, this instrument has been predominantly used to detect fluxes of biogenic trace gases emitted by large forest-type ecosystems, and to understand mechanisms of biogenic volatile organic compounds (BVOC) formation in plants, as well as BVOC role in plant protection against abiotic and biotic stresses.

### 5.1. Atmospheric Chemistry

Plants emit in the atmosphere a suite of reactive gases, mainly belonging to the multifaceted family of biogenic volatile organic compounds (BVOC). Few among the most abundant BVOC emitted by vegetation, principally volatile isoprenoids such as isoprene, monoterpenes and sesquiterpenes, are important in atmospheric chemistry, as they may enter and catalyse the complex atmospheric reaction chains. In particular, volatile isoprenoids can (a) lead, in the presence of anthropogenic pollutants, especially NOx, to the formation of ozone [90]; (b) produce, again by reaction with pollutants, secondary organic aerosol [91]; (c) be therefore implicated in the ancillary effects caused by photochemical smog, namely absorption of specific solar wavelength and light scattering, and contribution to greenhouse gas accumulation directly (e.g., through ozone accumulation) or indirectly (e.g., through fast removal of reactive OH [92], and consequent accumulation of more slowly reactive greenhouse gases such as CH_4_ and CO_2_; (d) inhibit new particle formation [93]; and (e) be oxidized and then deposited into vegetation, and therefore cleansed without additional biogeochemical inputs [94].

In Italy, main advances about plant contribution of reactive trace gases to the atmosphere were the findings that: (a) Mediterranean forest plant species can emit a variegated spectrum of constitutive BVOC, by far more complex than the simple emission of isoprene which still seems to constitute the main and more abundant BVOC in non-Mediterranean forest ecosystems. The discovery of monoterpene-emitting oaks [95] is perhaps the most remarkable observation of chemical isoprenoids diversity in the Mediterranean, with subsequent observations further clarifying the complex links between isoprenoids emissions and taxonomy of oaks [96]. Monoterpene emitters are known to contribute less than isoprene to ozone formation, being rather involved in SOA and particle formation [97]; hence the importance of these observations for atmospheric chemistry; (b) future levels of CO_2_ are likely to uncouple isoprenoid emissions from photosynthesis. This is a surprising result since volatile isoprenoids are synthesized in the chloroplast from photosynthetic carbon [98]. Studies conducted in the CO_2_ springs in central Italy, where CO_2_ concentration attains superambient levels especially during the night, matched those simultaneously conducted with artificial CO_2_ enhancement [99], and clearly indicated an ubiquitous occurrence of the inhibition of isoprene emission in plants grown at elevated CO_2_[100]. The impact of CO_2_ might have a dramatic effect on future isoprene emissions at global level, attenuating or even reverting the build up of this trace gas in the atmosphere, and the consequent feedback on air chemistry [101].

The advent of PTR-MS technology, allowing fast and sensitive measurements, has made it possible to measure fluxes of BVOC out of canopies of whole ecosystems, rather than concentrations out of small foliar surfaces. This has contributed enormously to upscale measurements to large communities such as forests or crops. Results have substantially confirmed the importance of monoterpene-emitters in the Mediterranean natural vegetation, whereas isoprene emitters seem to play a substantial lower role as compared to more boreal or tropical vegetation types [102]. Several other important findings can be attributed to the use of PTR-MS in experiments to assess biosphere-atmosphere exchanges of gases: (a) the large and sustained flux of methanol from vegetation at certain phenological periods [103], especially in coincidence with rapid growth and cell wall loosening [104]; (b) the large but transient flux of green leaf volatiles from wounded surfaces [103], especially in coincidence with mechanical harvesting of grasses [104]; and (c) the appearance of compounds deriving from isoprene oxidation (methyl vinyl ketone and metacrolein) above canopies when high temperatures made more likely isoprene oxidation by reactive oxygen species also produced by leaves [105].

Recent evolution of PTR-TOF-MS, allowing quasi-instantaneous determination of the entire spectrum of volatiles, and better separation of isomers, is expected to further contribute to the determination of net fluxes of reactive trace gases by vegetation, especially when transient, as in the case of stress events [104] or in the case of tracers of primary productivity such as carbonyl sulphide (COS) [106].

### 5.2. Plant Biology

BVOC can be directly emitted or stored into specialized organs. Volatile isoprenoids are generally stored, but the simplest of them, isoprene, is never stored, reaching a global emission rate higher than the sum of all other isoprenoids, and accounts generally for 1%–2% of the photosynthetic carbon [107]. Stored isoprenoids reach concentrations that are auto-toxic, and are also toxic for herbivores and other plant enemies. They are therefore important components in plant defence strategies, functioning as repellents [108], sealing wounding [109], and attracting herbivore enemies in tritrophic or multitrophic interactions [110,111]. Isoprene may also interfere with insect feeding [112] but this does not seem to occur in all cases [113]. A handful of monoterpenes can also be emitted without being stored in specialized organs, but this seems to occur only in few Mediterranean oaks, and emissions are an order of magnitude lower than for isoprene [114].

Research in Italy has concentrated over the years on unveiling whether isoprene has a functional role. Pioneering experiments carried out in USA indicated that isoprene may protect leaves against thermal stresses [115]. However, the Italian teams found isoprene to protect against oxidative stresses [116], quenching reactive oxygen species [117] and reactive nitrogen species [118]. While these researches has contributed to the now unambiguously accepted notion that isoprene protects plants against abiotic stresses, the mechanisms by which isoprene emitters are stress-resistant are widely debated yet [119]. PTR-MS measurements, assisted by other biophysical techniques, revealed that emission of isoprene makes more stable thylakoid membranes in the chloroplasts [120], thus preserving the functionality of the photosynthetic apparatus, and in turn avoiding the appearance of reactive oxygen species that are formed when photosynthesis cannot make full use of light energy [121].

A second field of plant biology in which PTR-MS has allowed unprecedented progress is the determination of the biochemical pathway of isoprene and monoterpene formation. By using quasi-continuous detection of labelled compounds, it is indeed possible to follow the time of course of volatile isoprenoids labelling [122]. This has confirmed and further refined seminal experiments showing labelling time-courses of isoprene [123] and monoterpenes [114] compatible with direct shunting of photosynthetic carbon into these families of volatile isoprenoids, and thus implicating synthesis of isoprenoids in the chloroplasts [119]. Perhaps even more interestingly, PTR-MS has allowed scientists to unravel that different sources of carbon may start being used to form isoprene, especially when leaves cannot use photosynthetic carbon because of concurrent strong stress events, namely drought [122]. This is an important and replicable observation, as it speaks for the importance of volatile isoprenoids in plant economy. Indeed, plants sustain the synthesis of isoprenoids even when photosynthesis is inhibited and cannot contribute carbon anymore, making the overall carbon budget negative, but possibly achieving the desired protection against photoinhibition and photooxidation.

### 5.3. Air Quality Monitoring: PTR-MS and Olfactometry

Besides its already-mentioned importance in aroma profiling, sensory analysis can also be used to estimate outdoor and indoor air quality and in particular odour emissions from industrial plants. The latter has a great impact on the everyday life of people living in the neighbourhood of the source as well as on compliance with law regulations. From a toxicological point of view, specific laws set limits in the permitted amount for few known substances. However, odour perception by human beings is a complex process, still not fully understood and producing nonlinear responses to VOC concentrations [124]. Concentration thresholds set by law are often far above odour perception concentration. The golden standard to assess the impact on human beings of odours is olfactometry, which has also been introduced in the legislation of some countries. Olfactometry directly measures the human response to odour stimuli employing specifically trained people. The evident drawbacks are the expensiveness, the difficulty of continuous monitoring and the impossibility to measure harmful compounds. Several alternatives to olfactometry have been attempted. On the one hand, techniques based on gas chromatography lack simplicity and more importantly rapidity [125]. On the other hand, fast and cheap electronic noses often lack sensitivity and show poor correlation with corresponding olfactometric data [126,127]. At the contrary, strong correlations between olfactometric assessments and PTR-MS intensity signals for various situations related to composting plants for municipal solid waste management (waste storage, waste management, biofiltering) have been reported [128] and confirmed in following studies [129]. Moreover multivariate calibration methods such as PLS effectively predict olfactory intensity of unknown samples on the sole basis of fast PTR-MS fingerprints [128].

**Figure 6.**
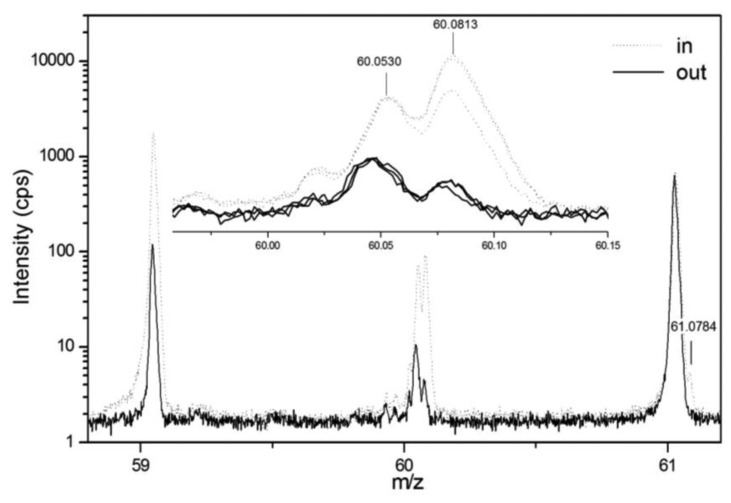
Excerpt of the PTR-TOF-MS spectra of six samples collected upstream (dotted lines) and downstream (continuous lines) a biofilter. The upper panel is a detail of the peak at *m*/*z* = 60. Numbers indicate the expected exact mass of protonated monosubstituted acetone isotope (60.053) and of protonated trimethylamine (60.0813). At mass 61.0784 the mono substituted isotope of trimethylamine is also indicated. Reprinted from [129], with permission from the copyright holders, IWA Publishing.

Within the same studies inlet and outlet air of biofilters have been investigated. The marked differences for several PTR-MS peaks provide a preliminary indication of possible markers of biofilter efficiency and the feasibility of *in situ* continuous monitoring [128,129]. PTR-Quad-MS provides limited compound separation, given the unit mass resolution. To partially overcome this problem, the use of PTR-ToF-MS has been attempted [129]. This enables to resolve the structure of nominal mass peaks, thus disentangling the contribution of isobaric compounds, such as for example acetone ^13^C-isotope and trimethylammine at *m*/*z* = 60 (Figure 6).

## 6. Italian PTR-MS Applications in Medical Science

Medicine has always used breath analysis as an empirical diagnostic method to detect some diseases. Thanks to the introduction of analytical instrumentations, starting from the early chromatographic studies carried out in the 70s [130], thousands of volatile compounds have been identified in the human breath. Modern medical science builds on this observation to find potential markers of diseases. Breath analysis may constitute an easy and powerful tool for medical diagnostics with several evident advantages including excellent acceptance by patients since it is non-invasive and painless. Several breath tests have been introduced [131] but the full potential of this approach is yet to be unveiled. Recently direct injection mass spectrometry is being exploited in this field and some markers have been proposed [131], although difficulties in data interpretation and the high inter-individual variability often leave many open questions on their biochemical origin and reliability.

PTR-ToF-MS is a very promising tool for breath analysis and very recently the first Italian contribution in this sense has been published [20]. Aprea *et al.* studied the breath of sixteen rats with different dietary regimes. It is common to use animal models in order to investigate the mechanisms underlying specific diseases and in particular the influence of the dietary regime on the volatile content of breath can be studied. The rats under investigation were divided into four groups differing in the dietary regime (standard or high fat diet) or in the drinks supplied (water or decaffeinated coffee). Some volatiles differing in breath concentration between the rat groups were identified. For instance methanol, sulphur compounds including dimethyl sulphide and dimethyl sulphone, and ammonia differentiated standard and high fat dietary regimes, thus providing indications of VOCs possibly linked to the liver metabolism. Moreover higher concentrations of compounds directly related to coffee were detected in the breath of rats drinking decaffeinated coffee.

More recently FEM and the University of Naples joined in a project to evaluate the diagnostic potential of PTR-ToF-MS in the case of liver diseases. It was indeed possible to identify a correlation between several peaks identified in the breath of patients and the different stage of cirrhosis and, more interestingly it was possible to correlate them with some compounds measured in blood [21].

## 7. Conclusions and Perspectives

PTR-MS is a high sensitivity, rapid and non-invasive “sensor” which can be used for the monitoring of volatile compounds. It has been successfully applied to a variety of fields including environmental science, food science and technology, plant physiology and medical science. In some cases PTR-MS has been used for the identification and quantification of relevant compounds but it can also be used as rapid highly specific MS-e-nose for the setting of classification or calibration models.

Italian scientists have provided important contributions in many issues related to the development and application of PTR-MS and further research is going in different laboratories, some of them having acquired their PTR-MS instruments only recently. The strength of these groups seems to be in food science and technology, plant physiology and environmental application although recently also bioprocess monitoring and health applications have been successfully addressed.

## Abbreviations

CTR: Charge Transfer Reaction
DIMS: Direct Injection-Mass Spectrometry
dPLS: discriminant Partial Least Square
GC-MS: Gas Chromatography-Mass Spectrometry
LASSO: Least Absolute Shrinkage and Selection Operator
LDA: Linear Discriminant Analysis
MS: Mass Spectrometry
PA: Proton Affinity
PDA: Penalised Discriminant Analysis
PLS: Partial Least Squares
PLSR: Partial Least Squares Regression
ppbv: parts per billion by volume
ppmv: parts per million by volume
pptv: parts per trillion by volume
PTR-MS: Proton Transfer Reaction-Mass Spectrometry
PTR-ToF-MS: Proton Transfer Reaction-Time of Flight-Mass Spectrometry
RF: Random Forest
RFE: Recursive Feature Elimination
SPME: Solid Phase Micro Extraction
SVM: Support Vector Machine
VOC: Volatile Organic Compound
ToF: Time-of-Flight

## References

[1] Brunner C., Szymczak W., Höllriegl V., Mörtl S., Oelmez H., Bergner A., Huber R.M., Hoeschen C., Oeh U. (2010). Discrimination of cancerous and non-cancerous cell lines by headspace-analysis with PTR-MS. Anal. Bioanal. Chem..

[2] Holm-Nielsen J.B., Lomborg C.J., Oleskowicz-Popiel P., Esbensen K.H. (2008). On-line near infrared monitoring of glycerol-boosted anaerobic digestion processes: Evaluation of process analytical technologies. Biotechnol. Bioeng..

[3] Biasioli F., Yeretzian C., Märk T.D., Dewulf J., van Langenhove H. (2011). Direct-injection mass spectrometry adds the time dimension to (B)VOC analysis. Trends Anal. Chem..

[4] Hansel A., Jordan A., Holzinger R., Prazeller P., Vogel W., Lindinger W. (1995). Proton transfer reaction mass spectrometry: On-line trace gas analysis at the ppb level. Int. J. Mass Spectrom. Ion Proc..

[5] Lindinger W., Hansel A., Jordan A. (1998). On-line monitoring of volatile organic compounds at pptv levels by means of proton-transfer-reaction mass spectrometry (PTR-MS) medical applications, food control and environmental research. Int. J. Mass Spectrom. Ion Proc..

[6] Munson M.S.B., Field F.H. (1966). Chemical ionization mass spectrometry. I. General introduction. J. Am. Chem. Soc..

[7] Fehsenfeld F.C., Schmeltekopf A.L., Ferguson E.E. (1966). Thermal energy ion—Neutral reaction rates. IV. Nitrogen ion charge-transfer reactions with CO and CO2. J. Chem. Phys..

[8] Ferguson E.E., Fehsenfeld F.C., Schmeltekopf A.L., Bates D.R., Estermann I. (1969). Flowing Afterglow Measurements of Ion-Neutral Reactions. Advances in Atomic and Molecular Physics.

[9] Jordan A., Haidacher S., Hanel G., Hartungen E., Herbig J., Märk L., Schottkowsky R., Seehauser H., Sulzer P. (2009). An online ultra-high sensitivity Proton-transfer-reaction mass-spectrometer combined with switchable reagent ion capability (PTR+SRI−MS). Int. J. Mass Spectrom..

[10] De Gouw J., Warneke C. (2007). Measurements of volatile organic compounds in the earths atmosphere using proton-transfer-reaction mass spectrometry. Mass Spectrom. Rev..

[11] Blake R., Monks P., Ellis A. (2009). Proton-transfer reaction mass spectrometry. Chem. Rev..

[12] Prazeller P., Palmer P.T., Boscaini E., Jobson T., Alexander M. (2003). Proton transfer reaction ion trap mass spectrometer. Rapid Commun. Mass Spectrom..

[13] Mielke L.H., Erickson D.E., McLuckey S.A., Műller M., Wisthaler A., Hansel A., Shepson P.B. (2008). Development of a proton-transfer reaction-linear ion trap mass spectrometer for quantitative determination of volatile organic compounds. Anal. Chem..

[14] Graus M., Müller M., Hansel A. (2010). High resolution PTR-TOF: Quantification and formula confirmation of VOC in real time. J. Am. Soc. Mass Spectrom..

[15] Sulzer P., Edtbauer A., Hartungen E., Jürschik S., Jordan A., Hanel G., Feil S., Jaksch S., Märk L., Märk T.D. (2012). From conventional proton-transfer-reaction mass spectrometry (PTR-MS) to universal trace gas analysis. Int. J. Mass Spectrom..

[16] Karl T., Hansel A., Cappellin L., Kaser L., Herdlinger-Blatt I., Jud W. (2012). Selective measurements of isoprene and 2-methyl-3-buten-2-ol based on NO^+^ ionization mass spectrometry. Atmos. Chem. Phys..

[17] Liu Y.J., Herdlinger-Blatt I., McKinney K.A., Martin S.T. (2012). Production of methyl vinyl ketone and methacrolein via the hydroperoxyl pathway of isoprene oxidation. Atmosph. Chem. Phys. Discuss.

[18] Boschetti A., Biasioli F., van Opbergen M., Warneke C., Jordan A., Holzinger R., Prazeller P., Karl T., Hansel A. (1999). PTR-MS real time monitoring of the emission of volatile organic compounds during postharvest aging of berryfruit. Postharvest Biol. Technol..

[19] Wisthaler A., Jensen N.R., Winterhalter R., Lindinger W., Hjorth J. (2001). Measurements of acetone and other gas phase product yields from the OH-initiated oxidation of terpenes by proton-transfer-reaction mass spectrometry (PTR-MS). Atmosph. Environ..

[20] Aprea E., Morisco F., Biasioli F., Vitaglione P., Cappellin L., Soukoulis C., Lembo V., Gasperi F., D'Argenio G., Fogliano V. (2012). Analysis of breath by proton transfer reaction time of flight mass spectrometry in rats with steatohepatitis induced by high-fat diet. J. Mass Spectrom..

[21] Morisco F., Aprea E., Lembo V., Fogliano V., Vitaglione P., Mazzone G., Cappellin L., Gasperi F., Masone S., De Palma G.D. (2013). Rapid “breath-print” of liver cirrhosis by proton transfer reaction time-of-flight mass spectrometry. A pilot study. PLoS One.

[22] Cappellin L., Probst M., Limtrakul J., Biasioli F., Schuhfried E., Soukoulis C., Märk T.D., Gasperi F. (2010). Proton transfer reaction rate coefficients between H_3_O^+^ and some sulphur compounds. Int. J. Mass Spectrom..

[23] Cappellin L., Karl T., Probst M., Ismailova O., Winkler P.M., Soukoulis C., Aprea E., Märk T.D., Gasperi F., Biasioli F. (2012). On quantitative determination of volatile organic compound concentrations using proton transfer reaction time-of-flight mass spectrometry. Environ. Sci. Technol..

[24] Cappellin L., Biasioli F., Fabris A., Schuhfried E., Soukoulis C., Mark T., Gasperi F. (2010). Improved mass accuracy in PTR-TOF-MS: Another step towards better compound identification in PTR-MS. Int. J. Mass Spectrom..

[25] Cappellin L., Aprea E., Granitto P., Wehrens R., Soukoulis C., Viola R., Märk T.D., Gasperi F., Biasioli F. (2012). Linking GC-MS and PTR-TOF-MS fingerprints of food samples. Chemom. Intell. Lab. Syst..

[26] Schuhfried E., Biasioli F., Aprea E., Cappellin L., Soukoulis C., Ferrigno A., Märk T.D., Gasperi F. (2011). PTR-MS measurements and analysis of models for the calculation of Henry's law constants of monosulfides and disulfides. Chemosphere.

[27] Cappellin L., Biasioli F., Schuhfried E., Soukoulis C., Märk T.D., Gasperi F. (2011). Extending the dynamic range of proton transfer reaction time-of-flight mass spectrometers by a novel dead time correction. Rapid Commun. Mass Spectrom..

[28] Cappellin L., Biasioli F., Granitto P., Schuhfried E., Soukoulis C., Märk T.D., Gasperi F. (2011). On data analysis in PTR-TOF-MS: From raw spectra to data mining. Sens. Actuators B.

[29] De Gouw J., Warneke C., Karl T., Eerdekens G., van der Veen C., Fall R. (2003). Sensitivity and specificity of atmospheric trace gas detection by proton-transfer-reaction mass spectrometry. Int. J. Mass Spectrom..

[30] Warneke C., van der Veen C., Luxembourg S., de Gouw J.A., Kok A. (2001). Measurements of benzene and toluene in ambient air using proton-transfer-reaction mass spectrometry: Calibration, humidity dependence, and field intercomparison. Int. J. Mass Spectrom..

[31] Španěl P., Smith D. (1995). Reactions of hydrated hydronium ions and hydrated hydroxide ions with some hydrocarbons and oxygen-bearing organic molecules. J. Phys. Chem..

[32] Tani A., Hayward S., Hewitt C.N. (2003). Measurement of monoterpenes and related compounds by proton transfer reaction-mass spectrometry (PTR-MS). Int. J. Mass Spectrom..

[33] Smith D., Španěl P. (2011). Direct, rapid quantitative analyses of BVOCs using SIFT-MS and PTR-MS obviating sample collection. Trends Anal. Chem..

[34] Von Hartungen E., Wisthaler A., Mikoviny T., Jaksch D., Boscaini E., Dunphy P., Mark T. (2004). Proton-transfer-reaction mass spectrometry (PTR-MS) of carboxylic acids—Determination of Henry's law constants and axillary odour investigations. Int. J. Mass Spectrom..

[35] Schuhfried E., Aprea E., Cappellin L., Soukoulis C., Viola R., Märk T.D., Gasperi F., Biasioli F. (2012). Desorption kinetics with PTR-MS: Isothermal differential desorption kinetics from a heterogeneous inlet surface at ambient pressure and a new concept for compound identification. Int. J. Mass Spectrom..

[36] Titzmann T., Graus M., Müller M., Hansel A., Ostermann A. (2010). Improved peak analysis of signals based on counting systems: Illustrated for proton-transfer-reaction time-of-flight mass spectrometry. Int. J. Mass Spectrom..

[37] Chernushevich I.V., Loboda A.V., Thomson B.A. (2001). An introduction to quadrupole-time-of-flight mass spectrometry. J. Mass Spectrom..

[38] Stephan T., Zehnpfenning J., Benninghoven A. (1994). Correction of dead-time effects in time-of-flight mass-spectrometry. J. Vac. Sci. Technol. A-Vac. Surf. Films.

[39] Coates P.B. (1992). Analytical corrections for dead time effects in the measurement of time-interval distributions. Rev. Sci. Instrum..

[40] Biasioli F., Gasperi F., Aprea E., Mott D., Boscaini E., Mayr D., Mark T. (2003). Coupling proton transfer reaction-mass spectrometry with linear discriminant analysis: A case study. J. Agric. Food Chem..

[41] Gasperi F., Gallerani G., Boschetti A., Biasioli F., Monetti A., Boscaini E., Jordan A., Lindinger W., Iannotta S. (2001). The mozzarella cheese flavour profile: A comparison between judge panel analysis and proton transfer reaction mass spectrometry. J. Sci. Food Agric..

[42] Granitto P., Biasioli F., Aprea E., Mott D., Furlanello C., Mark T., Gasperi F. (2007). Rapid and non-destructive identification of strawberry cultivars by direct PTR-MS headspace analysis and data mining techniques. Sens. Actuators B-Chem..

[43] Aprea E., Biasioli F., Gasperi F., Mott D., Marini F., Maerk T.D. (2007). Assessment of Trentingrana cheese ageing by proton transfer reaction-mass spectrometry and chemometrics. Int. Dairy J..

[44] Granitto P., Furlanello C., Biasioli F., Gasperi F. (2006). Recursive feature elimination with random forest for PTR-MS analysis of agroindustrial products. Chemom. Intell. Lab. Syst..

[45] Lindinger C., Labbe D., Pollien P., Rytz A., Juillerat M.A., Yeretzian C., Blank I. (2008). When machine tastes coffee: Instrumental approach to predict the sensory profile of espresso coffee. Anal. Chem..

[46] Biasioli F., Gasperi F., Yeretzian C., Märk T.D. (2011). PTR-MS monitoring of VOCs and BVOCs in food science and technology. Trends Anal. Chem..

[47] Simon J.E., Hetzroni A., Bordelon B., Miles G.E., Charles D.J. (1996). Electronic sensing of aromatic volatiles for quality sorting of blueberries. J. Food Sci..

[48] Aprea E., Biasioli F., Sani G., Cantini C., Mark T., Gasperi F. (2006). Proton transfer reaction-mass spectrometry (PTR-MS) headspace analysis for rapid detection of oxidative alteration of olive oil. J. Agric. Food Chem..

[49] Aprea E., Biasioli F., Sani G., Cantini C., Mark T., Gasperi F. (2008). Online monitoring of olive oil headspace by proton transfer reaction-mass spectrometry. Rivista italiana della sostanze grasse.

[50] Biasioli F., Gasperi F., Aprea E., Colato L., Boscaini E., Mark T. (2003). Fingerprinting mass spectrometry by PTR-MS: Heat treatment *vs.* pressure treatment of red orange juice—A case study. Int. J. Mass Spectrom..

[51] Gasperi F., Aprea E., Biasioli F., Carlin S., Endrizzi I., Pirretti G., Spilimbergo S. (2009). Effects of supercritical CO_2_ and N_2_O pasteurisation on the quality of fresh apple juice. Food Chem..

[52] Aprea E., Biasioli F., Carlin S., Endrizzi I., Gasperi F. (2009). Investigation of volatile compounds in two raspberry cultivars by two headspace techniques: Solid-phase microextraction/gas chromatography-mass spectrometry (SPME/GC-MS) and proton-transfer reaction-mass spectrometry (PTR-MS). J. Agric. Food Chem..

[53] Cappellin L., Aprea E., Granitto P., Romano A., Gasperi F., Biasioli F. (2013). Multiclass methods in the analysis of metabolomic datasets: The example of raspberry cultivar volatile compounds detected by GC-MS and PTR-MS. Food Res. Int..

[54] Cappellin L., Soukoulis C., Aprea E., Granitto P., Dallabetta N., Costa F., Viola R., Märk T.D., Gasperi F., Biasioli F. (2012). PTR-ToF-MS and data mining methods: A new tool for fruit metabolomics. Metabolomics.

[55] Spitaler R., Araghipour N., Mikoviny T., Wisthaler A., Via J.D., Märk T.D. (2007). PTR-MS in enology: Advances in analytics and data analysis. Int. J. Mass Spectrom..

[56] Aprea E., Biasioli F., Carlin S., Versini G., Märk T.D., Gasperi F. (2007). Rapid white truffle headspace analysis by proton transfer reaction mass spectrometry and comparison with solid-phase microextraction coupled with gas chromatography/mass spectrometry. Rapid Commun. Mass Spectrom..

[57] Galle S.A., Koot A., Soukoulis C., Cappellin L., Biasioli F., Alewijn M., van Ruth S.M. (2011). Typicality and geographical origin markers of protected origin cheese from the netherlands revealed by PTR-MS. J. Agric. Food Chem..

[58] Boscaini E., van Ruth S., Biasioli F., Gasperi F., Märk T.D. (2003). Gas Chromatography-Olfactometry (GC-O) and proton transfer reaction−mass spectrometry (PTR-MS) analysis of the flavor profile of grana padano, parmigiano reggiano, and grana trentino cheeses. J. Agric. Food Chem..

[59] Biasioli F., Gasperi F., Aprea E., Endrizzi I., Framondino V., Marini F., Mott D., Mark T. (2006). Correlation of PTR-MS spectral fingerprints with sensory characterisation of flavour and odour profile of “Trentingrana” cheese. Food Qual. Prefer..

[60] Fabris A., Biasioli F., Granitto P.M., Aprea E., Cappellin L., Schuhfried E., Soukoulis C., Märk T.D., Gasperi F., Endrizzi I. (2010). PTR-TOF-MS and data-mining methods for rapid characterisation of agro-industrial samples: Influence of milk storage conditions on the volatile compounds profile of Trentingrana cheese. J. Mass Spectrom..

[61] Soukoulis C., Biasioli F., Aprea E., Schuhfried E., Cappellin L., Märk T.D., Gasperi F. (2012). PTR-TOF-MS analysis for influence of milk base supplementation on texture and headspace concentration of endogenous volatile compounds in yogurt. Food Bioprocess Technol..

[62] Sanchez del Pulgar J., Soukoulis C., Biasioli F., Cappellin L., Garcia C., Gasperi F., Granitto P., Maerk T.D., Piasentier E., Schuhfried E. (2011). Rapid characterization of dry cured ham produced following different PDOs by proton transfer reaction time of flight mass spectrometry (PTR-ToF-MS). Talanta.

[63] Sánchez Del Pulgar J., Soukoulis C., Carrapiso A.I., Cappellin L., Granitto P., Aprea E., Romano A., Gasperi F., Biasioli F. (2013). Effect of the pig rearing system on the final volatile profile of Iberian dry-cured ham as detected by PTR-ToF-MS. Meat Sci..

[64] Sánchez del Pulgar J., Carrapiso A.I., Reina R., Biasioli F., García C. (2013). Effect of IGF-II genotype and pig rearing system on the final characteristics of dry-cured Iberian hams. Meat Sci..

[65] Soukoulis C., Cappellin L., Aprea E., Costa F., Viola R., Märk T.D., Gasperi F., Biasioli F. (2012). PTR-ToF-MS, a novel, rapid, high sensitivity and non-invasive tool to monitor volatile compound release during fruit post-harvest storage: The case study of apple ripening. Food Bioprocess Technol..

[66] White P.J. (2002). Recent advances in fruit development and ripening: An overview. J. Exp. Bot..

[67] Defilippi B.G., Dandekar A.M., Kader A.A. (2005). Relationship of ethylene biosynthesis to volatile production, related enzymes, and precursor availability in apple peel and flesh tissues. J. Agric. Food Chem..

[68] Golding J.B., McGlasson W.B., Wyllie S.G. (2001). Relationship between production of ethylene and alpha-farnesene in apples, and how it is influenced by the timing of diphenylamine treatment. Postharvest Biol. Technol..

[69] Bourne M.C. (2002). Food Texture and Viscosity: Concept and Measurement.

[70] Zini E., Biasioli F., Gasperi F., Mott D., Aprea E., Märk T.D., Patocchi A., Gessler C., Komjanc M. (2005). QTL mapping of volatile compounds in ripe apples detected by proton transfer reaction-mass spectrometry. Euphytica.

[71] Costa F., Cappellin L., Zini E., Patocchi A., Kellerhals M., Komjanc M., Gessler C., Biasioli F. (2013). QTL validation and stability for Volatile Organic Compounds (VOCs) in apple. Plant Sci..

[72] Carbone F., Mourgues F., Biasioli F., Gasperi F., Mark T., Rosati C., Perrotta G. (2006). Development of molecular and biochemical tools to investigate fruit quality traits in strawberry elite genotypes. Mol. Breed..

[73] Aprea E., Biasioli F., Carlin S., Mark T., Gasperi F. (2008). Monitoring benzene formation from benzoate in model systems by proton transfer reaction-mass spectrometry. Int. J. Mass Spectrom..

[74] Soukoulis C., Aprea E., Biasioli F., Cappellin L., Schuhfried E., Märk T.D., Gasperi F. (2010). Proton transfer reaction time-of-flight mass spectrometry monitoring of the evolution of volatile compounds during lactic acid fermentation of milk. Rapid Commun. Mass Spectrom..

[75] Tamime A.Y., Robinson R.K. (2007). Tamime and Robinson's Yoghurt: Science and Technology.

[76] De Brabandere A.G., de Baerdemaeker J.G. (1999). Effects of process conditions on the pH development during yogurt fermentation. J. Food Eng..

[77] Papurello D., Soukoulis C., Schuhfried E., Cappellin L., Gasperi F., Silvestri S., Santarelli M., Biasioli F. (2012). Monitoring of volatile compound emissions during dry anaerobic digestion of the organic fraction of municipal solid waste by proton transfer reaction time-of-flight mass spectrometry. Bioresour. Technol..

[78] Lanzini A., Leone P. (2010). Experimental investigation of direct internal reforming of biogas in solid oxide fuel cells. Int. J. Hydrog. Energy.

[79] Mata-Alvarez J., Macé S., Llabrés P. (2000). Anaerobic digestion of organic solid wastes. An overview of research achievements and perspectives. Bioresour. Technol..

[80] Hagen A., Rasmussen J.F. B., Thyden K. (2011). Durability of solid oxide fuel cells using sulfur containing fuels. J. Power Sources.

[81] Taylor A.J., Linforth R.S. T. (2010). Food Flavour Technology.

[82] Aprea E., Biasioli F., Gasperi F., Märk T.D., van Ruth S. (2006). *In vivo* monitoring of strawberry flavour release from model custards: Effect of texture and oral processing. Flavour Fragr. J..

[83] Ting J.L.V., Soukoulis C., Silcock P., Cappellin L., Romano A., Aprea E., Bremer P.J., Märk T.D., Gasperi F., Biasioli F. (2012). *In Vitro* and *In Vivo* flavor release from intact and fresh-cut apple in relation with genetic, textural, and physicochemical parameters. J. Food Sci..

[84] Heenan S., Soukoulis C., Silcock P., Fabris A., Aprea E., Cappellin L., Märk T.D., Gasperi F., Biasioli F. (2012). PTR-TOF-MS monitoring of *in vitro* and *in vivo* flavour release in cereal bars with varying sugar composition. Food Chem..

[85] Karl T., Yeretzian C., Jordan A., Lindinger W. (2003). Dynamic measurements of partition coefficients using proton-transfer-reaction mass spectrometry (PTR–MS). Int. J. Mass Spectrom..

[86] Aprea E., Biasioli F., Mark T., Gasperi F. (2007). PTR-MS study of esters in water and water/ethanol solutions: Fragmentation patterns and partition coefficients. Int. J. Mass Spectrom..

[87] Burnett M.G. (1963). Determination of partition coefficients at infinite dilution by the gas chromatographic analysis of the vapor above dilute solutions. Anal. Chem..

[88] Pollien P., Jordan A., Lindinger W., Yeretzian C. (2003). Liquid-air partitioning of volatile compounds in coffee: Dynamic measurements using proton-transfer-reaction mass spectrometry. Int. J. Mass Spectrom..

[89] Tasin M., Cappellin L., Biasioli F. (2012). Fast direct injection mass-spectrometric characterization of stimuli for insect electrophysiology by proton transfer reaction-time of flight mass-spectrometry (PTR-ToF-MS). Sensors.

[90] Atkinson R. (2000). Atmospheric chemistry of VOCs and NOx. Atmosph. Environ..

[91] Kavouras I.G., Mihalopoulos N., Stephanou E.G. (1998). Formation of atmospheric particles from organic acids produced by forests. Nature.

[92] Carlo P.D., Brune W.H., Martinez M., Harder H., Lesher R., Ren X., Thornberry T., Carroll M.A., Young V., Shepson P.B. (2004). Missing OH reactivity in a forest: Evidence for unknown reactive biogenic VOCs. Science.

[93] Kiendler-Scharr A., Wildt J., Maso M.D., Hohaus T., Kleist E., Mentel T.F., Tillmann R., Uerlings R., Schurr U., Wahner A. (2009). New particle formation in forests inhibited by isoprene emissions. Nature.

[94] Karl T., Harley P., Emmons L., Thornton B., Guenther A., Basu C., Turnipseed A., Jardine K. (2010). Efficient atmospheric cleansing of oxidized organic trace gases by vegetation. Science.

[95] Loreto F., Ciccioli P., Cecinato A., Brancaleoni E., Frattoni M., Fabozzi C., Tricoli D. (1996). Evidence of the photosynthetic origin of monoterpenes emitted by quercus ilex L. Leaves by 13C labeling. Plant Physiol..

[96] Loreto F., Bagnoli F., Fineschi S. (2009). One species, many terpenes: Matching chemical and biological diversity. Trends Plant Sci..

[97] Schurgers G., Arneth A., Holzinger R., Goldstein A.H. (2009). Process-based modelling of biogenic monoterpene emissions combining production and release from storage. Atmos. Chem. Phys..

[98] Lichtenthaler H.K., Schwender J., Disch A., Rohmer M. (1997). Biosynthesis of isoprenoids in higher plant chloroplasts proceeds via a mevalonate-independent pathway. FEBS Lett..

[99] Rosenstiel T.N., Potosnak M.J., Griffin K.L., Fall R., Monson R.K. (2003). Increased CO_2_ uncouples growth from isoprene emission in an agriforest ecosystem. Nature.

[100] Scholefield P.A., Doick K.J., Herbert B.M. J., Hewitt C.N. S., Schnitzler J.-P., Pinelli P., Loreto F. (2004). Impact of rising CO_2_ on emissions of volatile organic compounds: Isoprene emission from Phragmites australis growing at elevated CO_2_ in a natural carbon dioxide spring. Plant Cell Environ..

[101] Arneth A., Schurgers G., Hickler T., Miller P.A. (2008). Effects of species composition, land surface cover, CO_2_ concentration and climate on isoprene emissions from European forests. Plant Biol..

[102] Davison B., Taipale R., Langford B., Misztal P., Fares S., Matteucci G., Loreto F., Cape N., Rinne J., Hewitt C.N. (2009). Concentrations and fluxes of biogenic volatile organic compounds above a Mediterranean macchia ecosystem in western Italy. Biogeosciences.

[103] Brilli F., Hörtnagl L., Bamberger I., Schnitzhofer R., Ruuskanen T.M., Hansel A., Loreto F., Wohlfahrt G. (2012). Qualitative and quantitative characterization of volatile organic compound emissions from cut grass. Environ. Sci. Technol..

[104] Loreto F., Barta C., Brilli F., Nogues I. (2006). On the induction of volatile organic compound emissions by plants as consequence of wounding or fluctuations of light and temperature. Plant Cell Environ..

[105] Jardine K.J., Monson R.K., Abrell L., Saleska S.R., Arneth A., Jardine A., Ishida F.Y., Serrano A.M. Y., Artaxo P., Karl T. (2012). Within-plant isoprene oxidation confirmed by direct emissions of oxidation products methyl vinyl ketone and methacrolein. Glob. Change Biol..

[106] Wohlfahrt G., Brilli F., HöRtnagl L., Xu X., Bingemer H., Hansel A., Loreto F. (2012). Carbonyl sulfide (COS) as a tracer for canopy photosynthesis, transpiration and stomatal conductance: Potential and limitations. Plant Cell Environ..

[107] Sharkey T.D., Yeh S. (2001). Isoprene emission from plants. Annu. Rev. Plant Physiol. Plant Mol. Biol..

[108] Bleeker P.M., Diergaarde P.J., Ament K., Guerra J., Weidner M., Schütz S., Both M.T.J.D., Haring M.A., Schuurink R.C. (2009). The role of specific tomato volatiles in tomato-whitefly interaction. Plant Physiol..

[109] Pasqua G., Monacelli B., Manfredini C., Loreto F., Perez G. (2002). The role of isoprenoid accumulation and oxidation in sealing wounded needles of Mediterranean pines. Plant Sci..

[110] Heil M. (2008). Indirect defence via tritrophic interactions. New Phytol..

[111] Fineschi S., Loreto F. (2012). Leaf volatile isoprenoids: An important defensive armament in forest tree species. iForest—Biogeosci. For..

[112] Loivamäki M., Mumm R., Dicke M., Schnitzler J.-P. (2008). Isoprene interferes with the attraction of bodyguards by herbaceous plants. PNAS.

[113] Brilli F., Ciccioli P., Frattoni M., Prestininzi M., Spanedda A.F., Loreto F. (2009). Constitutive and herbivore-induced monoterpenes emitted by Populus × euroamericana leaves are key volatiles that orient Chrysomela populi beetles. Plant Cell Environ..

[114] Loreto F., Ciccioli P., Brancaleoni E., Cecinato A., Frattoni M., Sharkey T.D. (1996). Different sources of reduced carbon contribute to form three classes of terpenoid emitted by Quercus ilex L. leaves. PNAS.

[115] Singsaas E.L., Lerdau M., Winter K., Sharkey T.D. (1997). Isoprene increases thermotolerance of isoprene-emitting species. Plant Physiol..

[116] Loreto F., Mannozzi M., Maris C., Nascetti P., Ferranti F., Pasqualini S. (2001). Ozone quenching properties of isoprene and its antioxidant role in leaves. Plant Physiol..

[117] Loreto F., Velikova V. (2001). Isoprene produced by leaves protects the photosynthetic apparatus against ozone damage, quenches ozone products, and reduces lipid peroxidation of cellular membranes. Plant Physiol..

[118] Velikova V., Pinelli P., Pasqualini S., Reale L., Ferranti F., Loreto F. (2005). Isoprene decreases the concentration of nitric oxide in leaves exposed to elevated ozone. New Phytol..

[119] Loreto F., Schnitzler J.-P. (2010). Abiotic stresses and induced BVOCs. Trends Plant Sci..

[120] Velikova V., Varkonyi Z., Szabo M., Maslenkova L., Nogues I., Kovacs L., Peeva V., Busheva M., Garab G., Sharkey T.D. (2011). Increased thermostability of thylakoid membranes in isoprene-emitting leaves probed with three biophysical techniques. Plant Physiol..

[121] Velikova V., Sharkey T.D., Loreto F. (2012). Stabilization of thylakoid membranes in isoprene-emitting plants reduces formation of reactive oxygen species. Plant Signal Behav..

[122] Brilli F., Barta C., Fortunati A., Lerdau M., Loreto F., Centritto M. (2007). Response of isoprene emission and carbon metabolism to drought in white poplar (*Populus alba*) saplings. New Phytol..

[123] Sharkey T.D., Wiberley A.E., Donohue A.R. (2008). Isoprene emission from plants: Why and how. Ann. Bot..

[124] Stoddart D.M. (1990). The Scented Ape: The Biology and Culture of Human Odour.

[125] Defoer N., De Bo I., van Langenhove H., Dewulf J., van Elst T. (2002). Gas chromatography–mass spectrometry as a tool for estimating odour concentrations of biofilter effluents at aerobic composting and rendering plants. J. Chromatogr. A.

[126] Nicolas J., Romain A.-C., Wiertz V., Maternova J., André P. (2000). Using the classification model of an electronic nose to assign unknown malodours to environmental sources and to monitor them continuously. Sens. Actuators B Chem..

[127] Stuetz R.M., Fenner R.A., Engin G. (1999). Assessment of odours from sewage treatment works by an electronic nose, H2S analysis and olfactometry. Water Res..

[128] Biasioli F., Gasperi F., Odorizzi G., Aprea E., Mott D., Marini F., Autiero G., Rotondo G., Mark T. (2004). PTR-MS monitoring of odour emissions from composting plants. Int. J. Mass Spectrom..

[129] Biasioli F., Aprea E., Gasperi F., Mark T. (2009). Measuring odour emission and biofilter efficiency in composting plants by proton transfer reaction-mass spectrometry. Water Sci. Technol..

[130] Pauling L., Robinson A.B., Teranishi R., Cary P. (1971). Quantitative analysis of urine vapor and breath by gas-liquid partition chromatography. Proc. Natl. Acad. Sci. USA.

[131] Amann A., Smith D. (2005). Breath Analysis for Clinical Diagnosis and Therapeutic Monitoring.

